# Opposite Reactivity of Meningeal versus Cortical Microvessels to the Nitric Oxide Donor Glyceryl Trinitrate Evaluated *In Vivo* with Two-Photon Imaging

**DOI:** 10.1371/journal.pone.0089699

**Published:** 2014-02-28

**Authors:** Evgeny Pryazhnikov, Mikhail Kislin, Marina Tibeykina, Dmytro Toptunov, Anna Ptukha, Artem Shatillo, Olli Gröhn, Rashid Giniatullin, Leonard Khiroug

**Affiliations:** 1 Neuroscience Center, University of Helsinki, Helsinki, Finland; 2 Neurotar LTD, Helsinki, Finland; 3 A. I. Virtanen Institute for Molecular Sciences, University of Eastern Finland, Kuopio, Finland; Scuola Superiore Sant'Anna, Italy

## Abstract

Vascular changes underlying headache in migraine patients induced by Glyceryl trinitrate (GTN) were previously studied with various imaging techniques. Despite the long history of medical and experimental use of GTN, its effects on the brain vasculature are still poorly understood presumably due to low spatial resolution of the imaging modalities used so far. We took advantage of the micrometer-scale vertical resolution of two-photon microscopy to differentiate between the vasodynamic effects of GTN on meningeal versus cortical vessels imaged simultaneously in anesthetized rats through either thinned skull or glass-sealed cranial window. Intermediate and small calibre vessels were visualized *in vivo* by imaging intravascular fluorescent dextran, and detection of blood flow direction allowed identification of individual arterioles and venules. We found that i.p.-injected GTN induced a transient constriction of meningeal arterioles, while their cortical counterparts were, in contrast, dilated. These opposing effects of GTN were restricted to arterioles, whereas the effects on venules were insignificant. Interestingly, the NO synthase inhibitor L-NAME did not affect the diameter of meningeal vessels but induced a constriction of cortical vessels. The different cellular environment in cortex versus meninges as well as distinct vessel wall anatomical features probably play crucial role in the observed phenomena. These findings highlight differential region- and vessel-type-specific effects of GTN on cranial vessels, and may implicate new vascular mechanisms of NO-mediated primary headaches.

## Introduction

Vascular headaches were traditionally associated with abnormal changes in blood flow in the intracranial vessels [1], and nowadays the role of vessels in primary or secondary headaches remains a matter of continuous debate [2], [3], [4], [5], [6], [7]. In order to obtain novel information on intracranial vascular processes during headache, a variety of methods have been employed, ranging from transcranial Doppler (TCD) [8] to high resolution magnetic resonance angiography (MRA) [6]. In animal models, closed cranial window with intravital epifluorescence microscopy is one of the best established methods since the end of 90s [9]. Both in human and animal studies, observational methods are often complemented with a triggering method, commonly the administration of the NO donor glyceryltrinitrate (GTN) [10], [11], [12], [13]. This medicine is known for its ability to trigger headaches in healthy subjects and to initiate migraine attacks in patients [11], [14], [15]. One important advantage of the GTN-triggered headache model is the temporal control of events following this drug administration. Thus, there are two phases of the GTN-induced headache: the initial pain starts immediately after GNT infusion and persists only for about 30 min, whereas the secondary migraine-like pain develops after several hours [10], [16]. Notably, headache is also the main side effect of GTN in cardiology, where this medicine is widely used to counteract the ischemic conditions.

Vasodilation of large intracranial cerebral arteries in response to GTN was shown in human volunteers using transcranial Doppler ultrasonography and single-photon emission computed tomography (SPECT) based on measurements of the cerebral blood flow [17]. Likewise, it has been shown in monkeys, using angiography, that the i.v. infusion of the GTN induced vasodilation of the middle cerebral artery (MCA) [18]. Using laser Doppler flowmetry (LDF), the dilatory effects of GTN on both MCA and the middle meningeal artery (MMA) have been reported [19]. Using 3T MRI, a transient vasodilatation (by up to 30%) of both large arteries has also been detected immediately after infusion of the GNT [3]. For all these methods, the main limiting factor is their poor spatial resolution that restricts the measurements to only large vessels such as MMA or MCA, while leaving the dynamics of smaller vessels beyond the reach of the techniques traditionally employed in vasculature imaging studies. Therefore, it remains unclear whether smaller vessels are dilated in response to GTN similarly to the large ones. Elucidating this issue is important, because small size meningeal vessels are innervated by caltitonin gene-related peptide containing nerves (CGRP) [20], which suggests that small vessel dynamics may play a role in migraine pathogenesis.

Already early studies showing differential sensitivity of proximal versus distal parts of the isolated middle cerebral artery to GTN [21] have indicated that the effects of GTN might be region specific. More recently, Greco and co-workers used LDF on anesthesized rats to demonstrate that GTN transiently reduced blood flow in dural vessels, but not in the cortical ones [22]. However, the limited spatial resolution of this technique made it impossible to distinguish between veins and arteries, and allowed only partial separation of meningeal and cortical signals.

In the present study, we explored the effects of GTN on the cerebral circulation with unprecedented temporal and spatial resolution by using two-photon laser scanning microscopy (TPLSM) in anesthetized rats, where vessels were visualized by an i.v. injection of FITC dextran. Unexpectedly, we found that meningeal (dural) arterioles were abruptly constricted, while cortical arterioles were dilated. In contrast to the previous reports of high sensitivity of large veins to GNT, we found that small venules were nearly insensitive to this NO donor.

## Materials and Methods

### Ethics Statement

All experiments on animals were performed with accordance to local guidance for animal care (The Finnish Act on Animal Experimentation (62/2006)). Animal license (ESAVI/2857/04.10.03/2012) from local authority (ELÄINKOELAUTAKUNTA-ELLA) was obtained and included all procedures used in this study.

### Animals and surgical procedures

Twenty seven Wistar rats ranging from 80 to 140 g in weight were used in this study (age range 21–35 days). The rats were kept in individual cages in the certified University’s animal facility and provided with food and water *ad libitum*. Only visually healthy, drug and test-naïve animals of both sexes were selected for experiments. Animals were randomly allocated to experimental groups. To address the RRR-policy requirements, the group size was selected based on the following criteria: i) normality of data distribution, ii) degree of data scatter (variability), iii) confidence level of 95% (P<0.05) as a target statistical significance. Every animal was used in only one imaging session and received only one experimental treatment. Rat was selected for this study because this species offers a more relevant animal model in terms of physiology of cerebral blood flow as compared to mice [23]. Furthermore, many earlier studies of the effects of GTN on cerebral vessels were performed on rats [9], [12], [13], [19], [22].

Rats were anaesthetized (i.p.) with mixture of ketamine (80 mg/kg) and xylazine (10 mg/kg). Ketamine/xylazine mixture was re-admininistured every hour if necessary at half-the-original dose. A subset of experiments was performed with urethane (1 g/kg i.p.). Body temperature was maintained using a heating pad. Arterial oxygen saturation and heart rate were monitored using a pulse oximeter (MouseOx, Starr Life Sciences, USA) attached to the rat’s hind paw. Parameters were constantly monitored to ensure that physiological state of the rat was stable. Unstable or non-adequate level of anaesthesia was one of criteria for termination of experiment and exclusion of the data from analysis.

For *in vivo* monitoring of meningeal and cortical blood vessel diameter, we used two approaches: implantation of cranial window [24] and skull thinning [25] (the protocols were adapted for rats). During preparation to surgery a subcutaneous injection of 0.1% lidocaine was used to reduce local pain at the incision site. For cranial window formation, a ∼3×3 mm round craniotomy was performed over the parietal cortex. Cranial bone was carefully removed, while *dura mater* remained intact. Great care was taken to minimize any possible damage to *dura mater*. Animals with damaged *dura mater* or haemorrhage were excluded from experiments and euthanized. The brain was then covered with sterile cortical buffer (125 mM NaCl, 5 mM KCl, 10 mM glucose, 10 mM HEPES, 2 mM CaCl_2_ and 2 mM MgSO_4_ in distilled H2O), and a 5 mm diameter No. 1.5 glass coverslip (Electron Microscopy Sciences, USA) was placed over the window and sealed using dental cement. For thinned skull preparation, we gently and carefully thinned a ∼2×2 mm region of the parietal skull until the bone thickness reached 30–50 µm. The main advantages of the open skull (cranial window) preparation are its relative simplicity, good signal-to-noise ratio and larger depth of imaging. The main disadvantage of this preparation is a relatively strong damaging effect on superficial meningeal and cortical tissues, which is especially apparent during the first post-surgery hours. Therefore, two-photon imaging sessions were performed after 2 days of recovery after cranial window implantation. In small subset of animals we observed development of local inflammation in the cranial window 2 days after window implantation: these animals were excluded from experiments and euthanized. In comparison to open skull, the thinned skull preparation minimizes the damage to the tissue but restricts the depth of optimal visualization. In this preparation, imaging was performed shortly after completing the surgery.

### Multiphoton imaging

Animals were imaged with the FV1000MPE two-photon microscope (Olympus, Japan) using the 25X water immersion high NA objective specially designed for in vivo two-photon imaging. Image stacks were collected with a vertical step of 2–3 µm. FITC dextran (MW = 2000 kDa) was pre-injected in the rat tail vein for visualization of brain vasculature. Fluorescence excitation was achieved with MaiTai femtosecond laser tuned to 800 nm. Emission light was collected using a band pass filter (515–560 nm). In every experiment one imaging field comprising 500 by 500 microns in xy axes was imaged. Using the cranial window preparation, in rats injected with FITC-dextran we were able to visualize the blood flow in small arteries and veins with the depth of penetration up to 600 µm below the cortical surface. Small stacks of images comprising both meningeal and cortical layers were acquired every 20–30 seconds. For better illustration purposes 4 neighbour timepoints were averaged to plot one point on the graph. We reconstructed 3D images of brain vasculature to verify our ability to distinguish between meningeal and cortical blood vessels. Reconstructions were done using Imaris software (Bitplane, Switzerland). For identification of vessel type (arterioles *versus* venules), we performed longitudinal line scans as described before [26]. Briefly, repetitive line scans along the axis of the vessels were acquired to form an image where moving erythrocytes produce streaks with a measurable slope. Using this slope, it was possible to determine the direction of blood flow and, by following the vessel branch bifurcations, deduce the type of each vessel (arteriole or venule).

### Drugs

We used intraperitoneal injection of GTN (Orion Pharma, Finland) in the dose of 10 mg/kg as described by Srikiatkhachorn *et al* [27] or Greco *et al* [22]. Since this solution contains also ethanol and propylene glycol, solution containing these compounds without GTN was used as a vehicle control. L-NAME (Sigma, USA) was delivered intraperitoneally (40 mg/kg) in PBS solution. FITC-conjugated dextran (MW = 2000 kDa, Sigma, USA) was dissolved in PBS to obtain a 5% solution and 150 µL were delivered intravenously in the tail vein for visualisation of blood vessels.

### Vessel diameter measurement

Measurements were done using the line profile function of ImageJ software. After manual drawing of the line across the selected segment of the vessel, line profile was plotted. Diameter was calculated as the average width at the half-height of the line profile plot. For measurement of diameter, we selected between 3 and 5 segments in different parts of one vascular element (i.e. we measured diameter changes in 3 to 5 different segments of a particular arteriole or venule). The segment was defined as the part of a vessel between two successive branching points or, if less than 3 branching points were present in the area of imaging, the regions for line profile analysis were selected at a distance of at least 100 µm from one to another. We then calculated the mean value by pooling the data from these segments, and used this mean value as the outcome of a particular experiment. This procedure ensured that each vascular element (e.g., a meningeal arteriole) from each particular animal contributed only once (n = 1) to the plots representing the diameter changes. Data were double-checked with automated “vessel diameter” plugin for ImageJ. The results from both methods were compared, and no significant differences in the outputs of the measurement methods were found.

### Blood pressure measurement

In the separate set of experiments blood pressure was measured in 4 male Wistar rats. Rats were initially anesthetized with Isoflurane (4% for induction 2% for maintenance) for the duration of surgical procedures (10 minutes). After canulation of right femoral artery with 24G catheter, anesthesia was switched to urethane (1 g/kg i.p.). The catheter was then connected to Datex Cardiocap II monitor (SOMA Technology Inc., Bloomfield, USA) through saline-filled pressure transducer for continuous blood pressure monitoring. After the surgery and switch of anesthesia, animals were allowed to recover for 15 minutes prior to recording. For statistical analysis values were taken every minute for 5 min before and 20 min after i.p. injection of GTN (10 mg/kg). Body temperature of the animals was maintained throughout the experiment using homeothermic blanket (Harvard Apparatus, Holliston, USA).

### Graphs and statistical analysis

Attempts were made to ensure blinded data analysis, by assigning the analysis tasks to those co-authors who did not participate in a particular imaging experiment. Graphs were plotted using Origin software (Microcal, USA). All graphs were normalized to the last two minutes preceding the drug administration. Images were acquired once every 20 or 30 seconds but for the better graphical presentation on graphs we averaged neighbour values to get one point every 2 minutes. For comparison of effects of GTN and other drugs on vessel diameter, we calculated mean area under the curve (AUC) for individual graphs plotted from original data. Namely, we used the “Integrate” tool of Origin to calculate area between the line drawn through the baseline (pre-injection) diameter level and the line delineating the changes in diameter from 0 to 16 minutes after drug injection. Thus, in case of vasoconstriction we obtained negative values of AUC (stronger constriction yielding more negative value) and in case of dilation - positive values. Statistical analysis was performed by EP and MK. The N values refer to the number of independent replications, each of them performed in a different animal. Data were presented as mean ± SEM. For statistical analyses using Student *t*-test, the P < 0.05 value was considered significant. Due to specific features of vascular architecture, in some of the experiments only specific types of vessels were visualized (i.e. only meningeal venules, cortical arterioles and cortical venules but not meningeal arterioles). This explains why N value may be not equal inside experimental group for different types of vessels.

## Results

### Visualization of meningeal and cortical vessels and distinguishing arteries from veins

**Figure 1:**
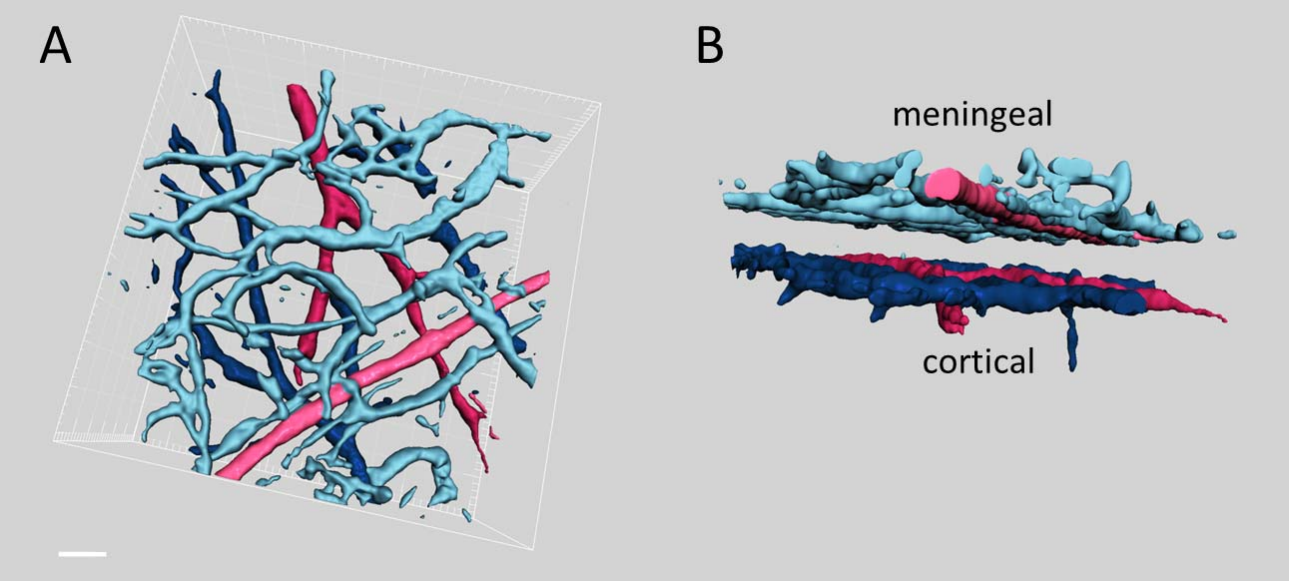
Upper (A) and side (B) views of 3D reconstruction of meningeal and cortical arterioles (red color) and venules (blue color) obtained from a typical z-stack of brain images. Note the clear gap between meningeal (dural) and cortical vessels in the side view projection shown (B). Scale bar 50 µm.

Acquisition of z-stacks in a time-lapse mode followed by the reconstruction of 3D images (Figure 1) allowed us to clearly distinguish meningeal blood vessels from the cortical vessels. Thus, Figure 1 (B) shows a distinct gap, i.e. the space between dural and cortical vessels. In order to distinguish arteries from veins, we performed longitudinal line scans and determined the direction of blood flow in each vessel of interest (see Methods for details). In summary, we optimized the high resolution tools for comparative analysis of the functional state of arterioles *versus* venules and of meningeal vessels *versus* cortical ones.

### Acute effects of GTN on vessel diameter

**Figure 2:**
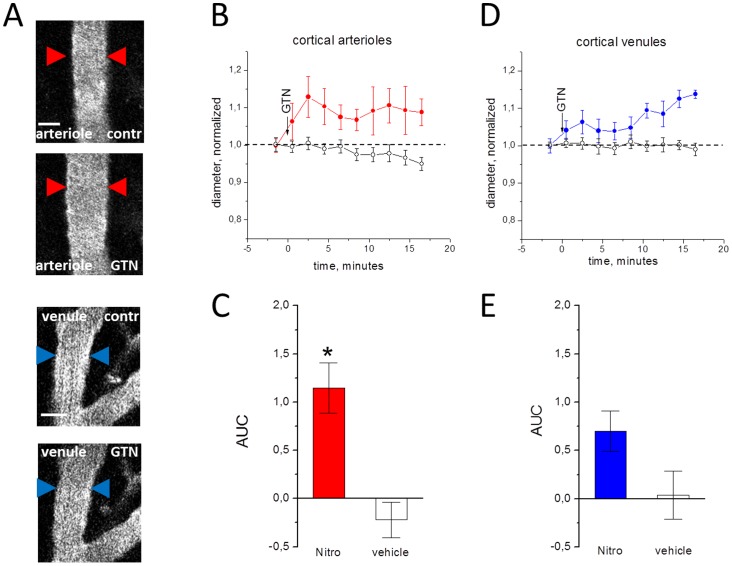
Effects of GTN on cortical vessels in the open cranial window preparation. A, examples of cortical vessels before (upper image) and after GTN administration (lower image). B, the normalized diameter of cortical arterioles after injection of the GTN (filled circles) or vehicle (empty circles), real diameter before application 36.1±6 µm for cortical arterioles and 38±8 µm for cortical venules. C, comparison of changes in the area under curve (AUC) in GTN versus vehicle in cortical arterioles (n = 6 and n = 3, respectively). D and E, the same for cortical venules (n = 8 and n = 4, respectively). Note that GTN significantly changed the diameter of arterioles (*  =  P<0.05) but not venules (P = 0.09). Scale bar 25 µm.

**Figure 3:**
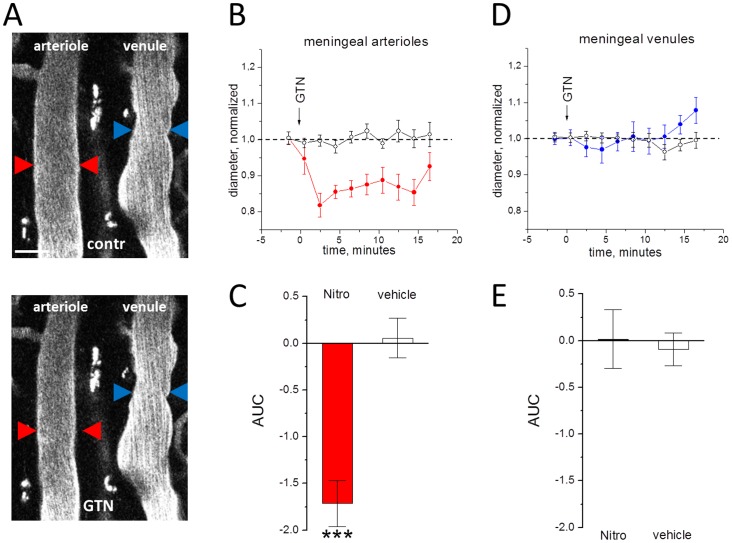
The action of of GTN on meningeal vessels in open cranial window preparation. **A**, examples of meningeal vessels before (upper image) and after GTN administration (lower image). **B**, the normalized diameter of meningeal arterioles after injection of the GTN (filled circles) or vehicle (empty circles), real diameter before application 17.4±2 µm for meningeal arterioles and 22.8±4 µm for meningeal venules. **C**, comparison of changes in the area under curve in GTN vs vehicle in meningeal arterioles (n = 6 and n = 4, respectively, ***  =  P<0.001), **D** and **E**, the same for meningeal venules (n = 5 and n = 4, respectively). Note that GTN changed the diameter of arterioles but not venules. Scale bar 40 µm.

The average diameter of investigated vessels was calculated as 17.4±2 µm for meningeal arterioles, 22.8±4 µm for meningeal venules, 36.1±6 µm for cortical arterioles and 38±8 µm for cortical venules. After injection of GTN there was a clear vasodilatatory effect on cortical arterioles (n = 6; P<0.05, Figure 2A,B,C) consistent with many previous observations [3], [17], [18], [19], [22]. There was also a clear trend for vasodilation of venules (Figure 2A,D,E) but this effect did not reach the significance (n = 8; P = 0.09). Interestingly, in arterioles the development of dilation was much faster than in venules (compare Figures 2B and 2D). Unexpectedly, in contrast to vasodilation of cortical vessels, GTN caused a significant vasoconstriction of meningeal arterioles (n = 6, P = 0.001, Figure 3A,B,C). This vasoconstriction of meningeal arterioles was transient and apparently subsided by 16 minutes (Figure 3B). No significant effects were observed in meningeal venules (n = 5; P = 0.61, Figure 3A,D,E).

Unlike GTN, injection of vehicle did not produce any significant changes in the diameter of all studied vascular regions (empty circles in Figures 2 and 3).

To verify independence of GTN effects from the type of preparation (i.e. open cranial window), similar experiments were performed on the thinned skull preparation. As shown in Figure S1, the effect of meningeal vasoconstriction was similar to the action of GTN in open cranial window preparation (p<0.01), although the dilation of cortical vessels was weaker in the case of thinned skull (p = 0.14 for cortical arterioles, Figure S1).

Since GTN is known to lower the systemic blood pressure [22], the observed vasoconstriction of meningeal arterioles may have resulted from a decrease in systemic blood pressure. We therefore asked whether in our experiments the injection of GTN produces changes in rat systemic blood pressure. We found that, within 15 s the GTN injection induced a drop in systolic blood pressure on average by 34% (n = 4, from 111.5±2.7 mmHg to 73.2 ±1.6 mmHg, Figure S2). The decreased blood pressure remained stable throughout the remaining 20 minutes of the measurement time.

Recent data indicate that, in the *in vivo* experiments, the type of general anaesthesia can determine the probability of migraine related cortical spreading depression [28] and responsiveness of astrocytes [29], which constitute a likely target of GTN-derived NO. In order to test the role of ketamine anaesthesia in the modulatory effects of GTN, we performed a subset of experiments with urethane (1 g/kg i.p.) As shown in Figure S3, change of anaesthesia type did not influence significantly the effects of GTN on meningeal arterioles which still constricted after peritoneal injection of this NO donor (n = 5, p<0.001). The lack of significant vasodilation in cortical arterioles under urethane anesthesia may be explained by the effect-modulating influence of urethane. Again, as in the case of thinned skull preparation, we observed a clear trend to cortical vasodilation in the urethane experiments.

Thus, in different types of anaesthesia (ketamine *versus* urethane) and experimental approaches for blood vessels visualization (open cranial window *versus* thinned skull) we consistently reproduced the main novel finding of early vasoconstriction of dural vessels in response to the NO donor GTN.

### The role of NO synthase blockade on the effects of GTN

**Figure 4:**
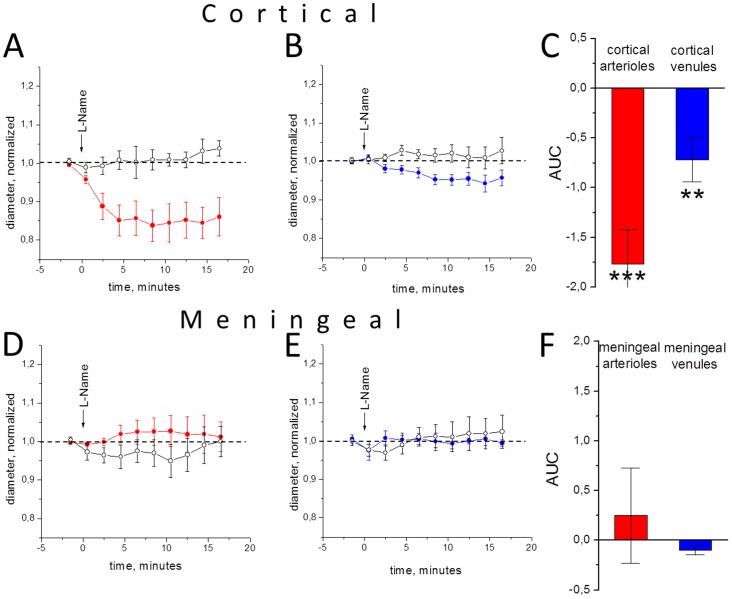
The action of L-NAME (filled circles) and vehicle (open circles) on the diameter of cortical and meningeal vessels in open cranial window preparation. A, the time of changes in cortical arterioles (real diameter 38.1±6 µm), B, cortical venules (real diameter 46.4±13 µm), D, meningeal arterioles (real diameter 20.7±5 µm) and E, meningeal venules (real diameter 24.1±7 µm), respectively. C and F, quantification of results (n = 9, 4, 8, 8 for meningeal arterioles, meningeal venules, cortical arterioles and cortical venules, respectively, *** = P<0.001, ** = P<0.01). Notice constriction of cortical but not of meningeal vessels.

To explore the role of NO in the differential action of GTN on meningeal and cortical vessels, we used the non-specific blocker of NO synthase L-NAME. Previously it has been shown that L-NAME increased systemic blood pressure after infusion in rats [30] likely due to the constriction of vessels. Consistent with this view, the i.p. injection of 40 mg/kg L-NAME induced vasoconstriction of cortical arterioles (n = 8; P<0.001, Figure 4A,B,C) and venules (n = 8; P<0.01). The action of L-NAME on meninges was highly variable, but on average the diameter of meningeal arterioles and venules changed little (n = 8; P = 0.47 and n = 4; P = 0.71, respectively, Figure 4 D,E,F). These results point to a key role of NO in control of blood flow in cortical but not meningeal vessels.

## Discussion

In this study we, for the first time, employed two-photon laser scanning microscopy (TPLSM) to visualize with high temporal and spatial resolution the acute effects of the GTN on meningeal (dural) versus cortical vessels. Using this technique, we found that cortical and dural vessels responded in opposing direction (with vasodilation *versus* vasoconstriction) to the same treatment with the NO donor GTN.

### The role of brain vessels in headache

In the classical theory of Woolf [1], vasodilation was for long time considered as the leading reason for primary headaches like migraine. Nevertheless, recent data indicated that headache could be eliminated by substances which do not show any vasoconstrictory effects, e.g. novel CGRP receptor antagonists [31]. Furthermore, substances like prostanoid PGF2α with strong vasoconstrictory effects do not produce headache in volunteers [5]. Involvement of vasculature in migraine pain pathogenesis is still actively debated [2], [3], [4], [6], [7]. Recent development of *in vivo* visualisation of blood vessels using multiphoton microscopy provides a powerful tool to address these issues with high resolution. Our present findings uncovered a previously unreported dichotomy in the reactions of intermediate diameter cortical *versus* dural vessels in the classical GTN model of headaches. These findings extend our knowledge about the complex behaviour of trigeminovascular system and, in particular, meningeal vessels innervated by trigeminal nerves where pain is likely generated.

### Methodological aspects

Multiphoton microscopy used in the current project has several advantages over the previously employed methods such as transcranial Doppler [8], single-photon emission computed tomography [17], angiography [6], [18] or intravital epifluorescent microscopy [9]. Thus, multiphoton imaging provides unprecedented high temporal and spatial resolutions of the vascular elements investigated in this study. Importantly, our approach allows clear distinction between different layers of brain vessels and unambiguous separation of *dura mater* vessels from the cortical ones without physical removal of *dura mater* (Figure 1). In our pilot experiments with removed *dura mater* we clearly observed cortical vessels with preserved morphology (Figure S4). Moreover, we found that cortical vessels dilate upon injection of GTN (Figure S4) similarly to the experiments with preserved *dura mater*. Such similarity indicated that the functional state of the cortical vessels was not significantly affected by the microsurgery. Furthermore, these pilot experiments suggested that GTN-induced cortical vasodilation develops independently from reflexes initiated from *dura mater* or diffusible agents released from that part of meninges.

In contrast to previously used methods, multiphoton imaging allows to study the diameter changes not only in large but also in mid-sized vessels, which present a large fraction of peripheral vascular bed. This is important because such the meningeal vessels of this calibre are innervated by CGRP expressing nerves [20]. Previously, by measuring venous distensibility in humans it has been observed that GTN dilates veins more so than arteries [32]. Here, we found that veins are relatively insensitive to GTN challenge. This could be related to the small diameter of the vessels visualized in our study and/or region-specific sensitivity of intracranial vessels to the NO donor. Indeed, the proximal parts of isolated middle cerebral artery have been previously shown to have a different sensitivity to GTN compared to the distal parts [21].

### Effect of GTN on meningeal vessels

Vasodilation of cortical vessels induced by GTN was observed by many other groups [3], [17], [18], [19], [22] and the findings of the present study are consistent with those observations. The more intriguing finding is the opposite, vasoconstrictory effect of GTN on meningeal (*dura mater*) vessels. Our findings with small diameter vessels supplement recent study employing intravital microscopy combined with LDF where it has shown a dilatory effect of GTN on large meningeal arteries [19]. Apart from different targets (microvessels versus large diameter arteries) there are several other obvious dissimilarities between our experimental approach and that of Gozalov and co-workers such as a difference in the concentration and route of GTN injection (20 µg/kg as i.v. bolus versus 10 mg/kg i. p. in our study). The issue of reliable animal migraine models (including the concentration of the trigger in GTN model) was recently discussed [33]. In our study we tried to minimize influence of several important parameters on outcome of the study: we tested GTN under two different anaesthesia methods, used vehicle with ethanol and propylene glycol as a control, compared two different access ways (cranial window and thinned skull). The insignificant effect of GTN in our study on cortical arterioles in the “thinned-skull” preparation can be explained by the fact that this preparation offers lower penetration depth and lower signal-to-noise ratio in the deeper layers (i.e., in the cortex) as compared to cranial window, whereas in the meninges variability (signal-to-noise ratio) is similar between thinned skull and cranial window.

Interestingly, Greco *et al* [22] using an LDF probe positioned in the meninges (somewhat above the cortical surface) found biphasic changes in the dural blood flow, which consisted in an initial fast decline followed by a progressive increase in the regional blood flow. The initial dip observed in that study could potentially be linked to the acute vasoconstriction we report here.

### Potential morphological and neurochemical mechanisms of differential reactivity of cortical and meningeal vessels

**Figure 5:**
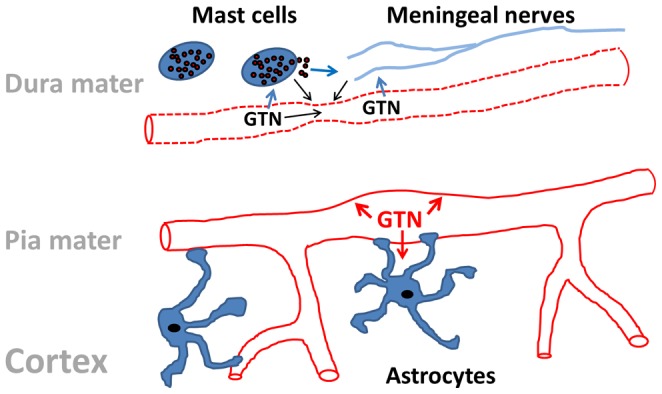
Schematic presentation of potential mechanisms for opposite modulation of dura mater and pial/cortical vessels by GTN. In *dura mater* occupied by mast cells and densely innervated by trigeminal and autonomous nerves GTN can induce vasoconstriction of small vessels either directly, or via release of vasoconstrictory agents from mast cells or through the neuronal control. The functional outcome depends on the combination of vasodilatatory versus vasoconstrictory agents and on the receptor profile. The dilatatory effect of GTN in pial/cortical vessels could be due to the direct action of this agent on the vessel wall or mediated via astrocytes releasing NO.

As illustrated schematically in Figure 5, there are several factors that may lead to the striking opposite reaction of cortical *versus* meningeal vessels to the very same treatment with GTN. Notably, there is an important difference in the local cellular environment regulating blood flow in cortex and in meninges: astrocytes control cortical (and pial) blood flow *via* their end feet that adhere to the vessel wall to form blood-brain barrier [34], [35], but such glial control is missing in *dura mater* vessels. Oppositely, *dura mater* is enriched with mast cells [36], and as GTN presumably degranulates mast cells [37], this could explain the vasoconstriction induced by this agent. Our preliminary unpublished data indicate strong vasoconstrictor effect in meninges induced by mast cell degranulating compounds (these pilot experiments will constitute the core of a future study). This additional vasoconstricting factor may overpower the direct vasodilatory effect of GTN in meningeal vessels, leading to the net decrease in meningeal artery diameter as reported here.

Vessel wall organization itself is also strikingly different between these vessel types: the walls of cortical vessels are part of the blood-brain barrier, which further includes endothelial cells with tight junctions and astrocytic end feet. Meningeal vessels, in contrast, lie inside the tight grooves of the cranium, and the walls of these vessels do not form the same kind of barrier as in cortical vessels. Interestingly, it has been suggested that, at least in humans, due to anatomical features the intracranial parts of dural meningeal artery cannot dilate [38], hence leaving room only for constriction in response to drugs or other stimuli.

In our study, we imaged small to medium-sized calibre vessels (for example, meningeal arterioles had the mean diameter of 17.4±2 µm). This is an important fact, because responses to compounds and stimulations may differ between proximal and distal parts of the meningeal and cortical vessels. It was shown, for example, that certain parts of the porcine meningeal artery are insensitive to the 5-hydroxytryptamine receptor agonist sumatriptan [39]. In another recent study, authors described layer-specific dilation of cortical penetrating arterioles to activation of the nucleus basalis of Meynert [40].

Some vasoactive substances, for example prostaglandins, can dilate or constrict vessels depending on type of vessels involved or molecular pathways involved [41]. Acetylcholine was shown to constrict simultaneously epicardial arteries and dilate smaller resistance vessels, depending on the interplay between direct vasoconstriction and endothelium-derived NO-mediated vasodilation [42].

From the clinical perspective our findings perhaps are more relevant not to the delayed (“migraine-like” headache) but rather to the early acute headache observed shortly after GTN administration [43]. One possible mechanism of this acute pain could be that the opposite vasodynamics in meningeal and cortical districts initiates a cascade of events leading to headache. Any direct action of NO on vessels may involve astrocytes, which control the blood flow in cortical areas, and mast cells, which surround not only dural vessels but also nociceptive nerve endings located in meninges [44]. Further studies are needed to clarify these potential mechanisms.

## Conclusion

In conclusion, we report here, for the first time, high resolution imaging of the effects of the headache-triggering agent GTN on medium-sized meningeal arteries and venules and compare them to cortical arteries and venules. We show that, in both regions, venules are essentially inert, whereas arterioles react to GTN in two opposing ways, i.e. by constriction in *dura mater* and dilation in the cortex. These data provide new insights on non-uniform vascular reactivity, which are important for understanding of the mechanisms of headache.

## Supporting Information

Figure S1 The action of GTN (filled circles) and vehicle (empty circles) on the diameter of cortical and meningeal arterioles (red) and venules (blue) in **thinned skull preparation**. A, the time of changes in cortical arterioles (real diameter 31.3±6 µm), B, cortical venules (real diameter 27.7±7 µm), D, meningeal arterioles (real diameter 22.5±3 µm) and E, meningeal venules (real diameter 23.6±5 µm), respectively. C and F, quantification of results (n = 3, 2, 4, 3 for meningeal arterioles, meningeal venules, cortical arterioles and cortical venules, respectively, ** = P<0.01).
(TIF)

Figure S2 The action of GTN on systemic systolic (filled circles) and diastolic (empty circles) blood pressure in rats (n = 4).
(TIF)

Figure S3 The action of GTN on the diameter of cortical and meningeal arterioles (red) and venules (blue) in open window preparation under **urethane** anaesthesia. A, the time of changes in cortical arterioles, B, cortical venules, D, meningeal arterioles and E, meningeal venules, respectively. C and F, quantification of results (n = 5, 5, 5, 4 for meningeal arterioles, meningeal venules, cortical arterioles and cortical venules, respectively, *** = P<0.001).
(TIF)

Figure S4 Cortical vessels in the *in vivo* imaging experiment with removed *dura mater*. 3D projection of cortical vessels in the *in vivo* imaging experiment with removed *dura mater*: A, upper view (look-through, or view from the top), B, side view of the same area (3D projection). Note the absence of dural vessels. Calibration bar – 15 µm. C, effect of GTN (i.p. injection, 10 mg/kg) on the diameter of cortical vessels. Note the transient vasodilation of cortical vessels in response to GTN.
(TIF)
