# Supplementary figures and images for: Opposite Reactivity of Meningeal versus Cortical Microvessels to the Nitric Oxide Donor Glyceryl Trinitrate Evaluated In Vivo with Two-Photon Imaging
Source: PLoS One. 2014 Feb 28;9(2):e89699. doi: 10.1371/journal.pone.0089699 (PMC3938546; doi:10.1371/journal.pone.0089699)

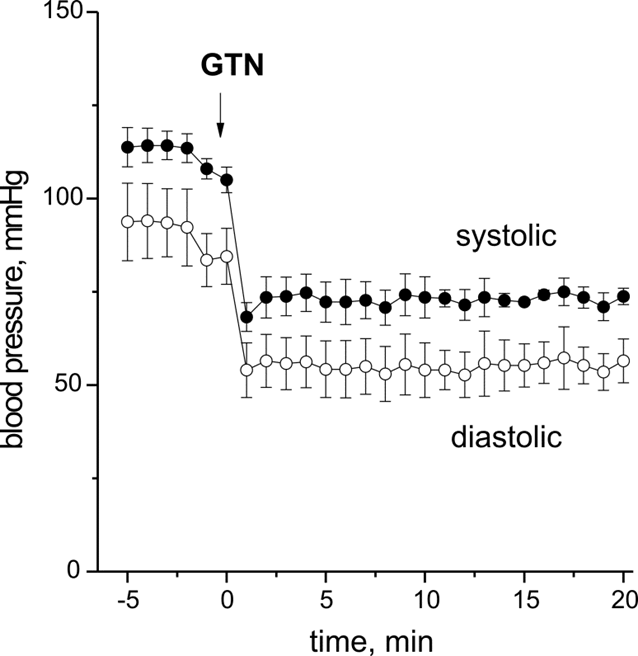

Supplement: Figure S2 — The action of GTN on systemic systolic (filled circles) and diastolic (empty circles) blood pressure in rats (n = 4). (TIF) [file pone.0089699.s002.tif]

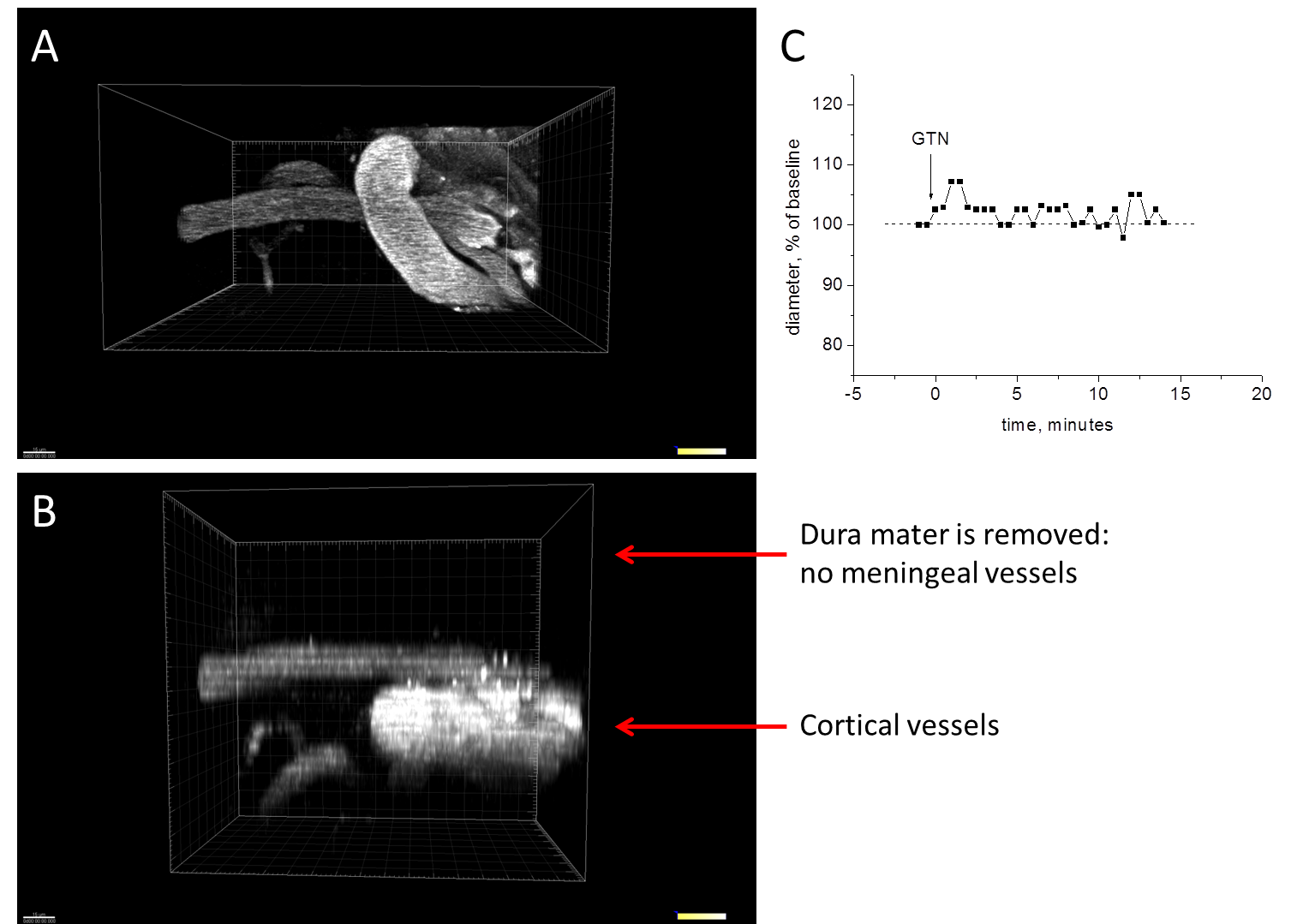

Supplement: Figure S4 — Cortical vessels in the in vivo imaging experiment with removed dura mater. 3D projection of cortical vessels in the in vivo imaging experiment with removed dura mater: A, upper view (look-through, or view from the top), B, side view of the same area (3D projection). Note the absence of dural vessels. Calibration bar – 15 µm. C, effect of GTN (i.p. injection, 10 mg/kg) on the diameter of cortical vessels. Note the transient vasodilation of cortical vessels in response to GTN. (TIF) [file pone.0089699.s004.tif]
